# Intradural *Staphylococcus aureus* Abscess of the *Cauda Equina* in an Otherwise Healthy Patient

**DOI:** 10.1155/2019/4860420

**Published:** 2019-01-22

**Authors:** Thomas J. Sorenson, Giuseppe Lanzino

**Affiliations:** ^1^Department of Neurologic Surgery, Mayo Clinic, Rochester, MN, USA; ^2^School of Medicine, University of Minnesota, Minneapolis, MN, USA; ^3^Department of Radiology, Mayo Clinic, Rochester, MN, USA

## Abstract

Abscesses involving the spine are usually located in the epidural space. In rare circumstances, intradural spinal abscesses can occur, typically in the setting of tuberculosis or other predisposing systemic conditions. In this illustrated case report, we discuss the imaging and intraoperative findings of an otherwise healthy patient with an intradural abscess of the *cauda equina* caused by *Staphylococcus aureus*. Although rare, intradural spinal abscesses can occur in the absence of typical “red flags” for infection, and a bacterial abscess should be considered in the differential diagnosis of intradural spinal cystic enhancing lesions.

## 1. Introduction

Abscesses involving the spine are typically localized to the epidural space. Intradural abscesses are quite rare and usually seen in the setting of tuberculosis or other predisposing systemic disease. However, although rare, an intradural abscess can be seen in an otherwise healthy patient in the absence of predisposing factors. In this case report, we discuss the imaging and intraoperative findings of an otherwise healthy patient with an intradural abscess of the *cauda equina* caused by *Staphylococcus aureus*. Although rare, intradural spinal abscesses can occur in the absence of typical “red flags” for infection, and a bacterial abscess should be considered in the differential diagnosis of intradural spinal cystic enhancing lesions, in addition to the consideration of myxopapillary ependymoma, hemangioblastoma, paraganglioma, cystic meningioma, spinal primitive neuroectodermal tumor (PNET), spontaneous or posttraumatic syringomyelia, dermoid or epidermoid cyst, and cystic dilatation of the *ventriculus terminalis*.

## 2. Case Description

### 2.1. History and Outside Examination and Imaging

**Figure 1:**
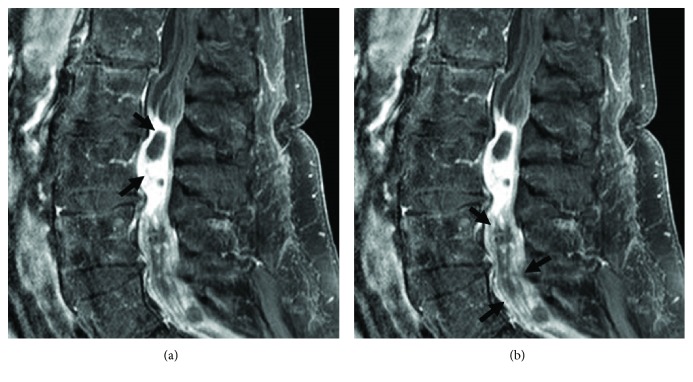
Magnetic resonance imaging (MRI) study was performed one month after onset of symptoms and showed (a) a lesion with extensive enhancement of the lesion periphery and (b) extension of enhancement to the distal nerve roots on sagittal fat-suppressed T1-weighted MRI.

**Figure 2:**
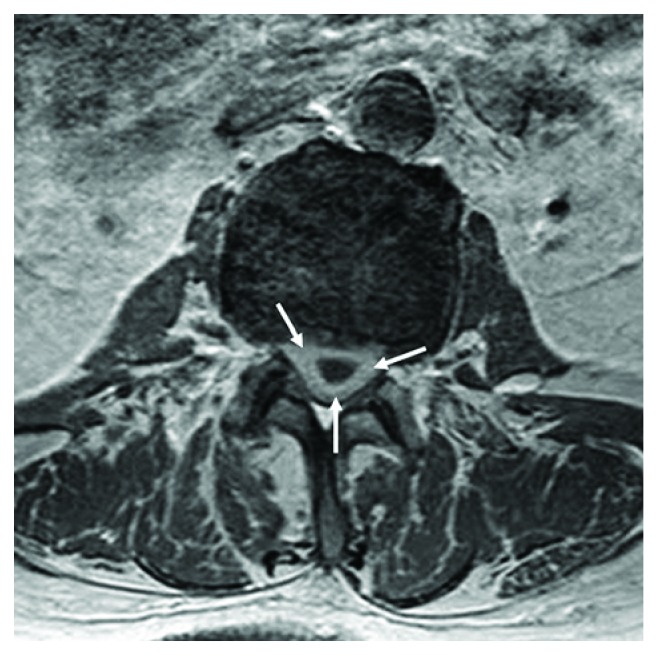
Axial T1-weighted MRI with contrast demonstrated hyperintense lesion periphery.

**Figure 3:**
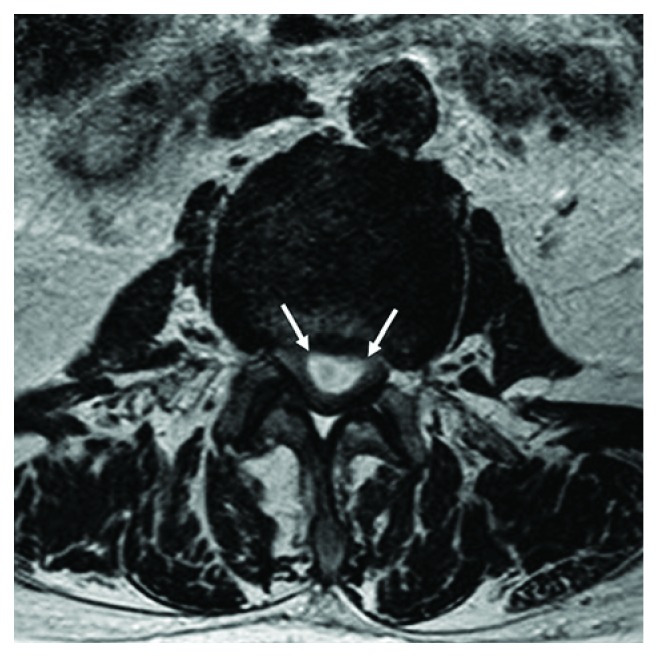
Axial T2-weighted MRI demonstrated homogenous hyperintensity of the lesion.

**Figure 4:**
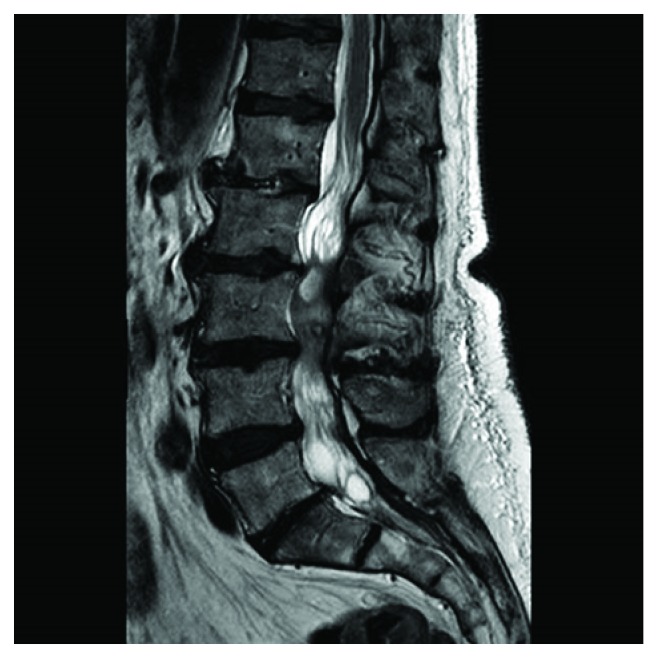
Sagittal T2-weighted MRI demonstrated evidence of degenerative changes but no involvement of the vertebral bodies or disc spaces.

An otherwise healthy 78-year-old man experienced subacute-onset back pain that radiated to both lower extremities and was worse with ambulation. Over the course of a month, the patient then experienced progressive neurological deterioration with bilateral leg weakness leading to an eventual inability to walk. Magnetic resonance imaging (MRI) study, performed one month after onset of symptoms, showed a lesion with extensive enhancement of the lesion periphery (Figure 1(a)) and extension of enhancement to the distal nerve roots on sagittal fat-suppressed T1-weighted MRI (Figure 1(b)). Axial T1-weighted MRI with contrast demonstrated hyperintense lesion periphery (Figure 2), and axial T2-weighted MRI demonstrated homogenous hyperintensity of the lesion (Figure 3). Lastly, sagittal T2-weighted MRI demonstrated evidence of degenerative changes but no involvement of the vertebral bodies or disc spaces (Figure 4). Since the patient did not have any indication of infection, our working diagnosis was that of a malignant neoplastic process with probable spread to the distal nerve roots.

### 2.2. Treatment and Perioperative Course

Based on the symptomatology and extensive involvement of the nerve roots, the plan was to perform a biopsy of the lesion to obtain a histological diagnosis. The patient was positioned prone on a Jackson table, and neuromonitoring for somatosensory evoked potentials (SSEP) and motor evoked potentials (MEP) was established (Supplementary Material 1). A vertical incision was made over L2 through L4, and intraoperative X-ray was used for localization of the lesion. Bilateral laminectomy of these levels was performed, and the dura was fully exposed and opened over the midline. At this point, thickened, reactive arachnoid was encountered. Opening the dura, we found that the nerve roots were plastered and very adherent to this arachnoid. In certain cases where the pathology is not immediately evident, intraoperative ultrasound is a useful adjunct to provide real-time confirmation of the location of the lesion. We have found this to be especially valuable in cases of intrinsic intramedullary lesions. With sharp dissection, an incision was made in this tissue, and the dissection was continued toward the surface of the mass. After incision of the capsule, thick puss was encountered. At this point, the true nature of the lesion became apparent, so pus was carefully drained, cultures were obtained, and the walls of the abscess were removed and biopsied. The dura was closed primarily with no observed changes in SSEPs or MEPs throughout the procedure. Cultures from the intraoperative specimen grew *S. aureus*. The patient experienced immediate improvement of pain after surgery and progressive, but incomplete, improvement of his weakness. He was discharged on postoperative day two with long-course intravenous antibiotics.

## 3. Discussion

Intradural extramedullary abscesses are rare lesions that have been infrequently reported [1–11]. The most commonly reported case was localized to the lumbar spine in patients in their 6th decade or later. Many of these cases are iatrogenic and arise after injection of local anesthetic, lumbar puncture, or discography [2]. *S. aureus* was the most commonly reported pathogen, and most cases are usually reported secondary to tuberculosis or other systemic conditions, including human immunodeficiency virus (HIV). The central nervous system represents an important target for HIV infection during multiple stages of the disease. This includes the (a) early phase, during which it acts as a viral reservoir after invasion of the host; (b) late phase, during which the CNS subverts its function and causes peripheral neuropathies and neurocognitive disorders; and (c) last phase, during which the CNS triggers opportunistic infections, cancers, and dementia [12]. Additionally, these lesions are typically diffusely located in the subdural space and not as a discrete mass lesion [2, 13, 14].

In these cases, clinical signs of infection in general are also often present. However, our case is unique in documenting the presence of an intradural abscess in a patient without any of these associated signs of systemic illness or predisposing systemic factors (e.g., diabetes mellitus). Thus, diagnosis of an intradural abscess should be considered in the differential diagnosis of a patient even without symptomatic illness or signs/symptoms of infection with the presence of an intradural mass on MRI with rapidly progressing symptoms.

In patients with *cauda equina* syndrome, surgical intervention for decompression and/or biopsy is always mandatory and should be performed in an expedited manner. For other patients with de novo intradural or intramedullary lesions, additional MRI studies may be warranted to better characterize the lesions by identifying signs of restriction on diffusion-weighted imaging (DWI), an axial gradient on region-of-interest (ROI), and constructive interference in steady state (CISS) sequence [15]. For these patients, it is only after extensive noninvasive imaging study is performed that a biopsy would be indicated as the most appropriate management option [16].

## 4. Conclusions

Even in otherwise healthy patients with *cauda equine* syndrome, intradural bacterial abscess should be considered as a possible differential diagnosis based on radiological findings of cystic enhancing lesions in this region.

## Supplementary Materials

Supplementary Materials Supplementary Material 1: illustrative intraoperative video of the case report.
